# A review of cardiovascular benefits of SGLT2 inhibitors

**DOI:** 10.1097/MD.0000000000030310

**Published:** 2022-09-09

**Authors:** Yingxia Zhang, Qinghua Han

**Affiliations:** a First Department of Clinical Medicine, Shanxi Medical University, Key Laboratory of Cellular Physiology at Shanxi Medical University, Ministry of Education, Shanxi Province, China; b Department of Cardiology, The 1st Hospital of Shanxi Medical University, Key Laboratory of Cellular Physiology at Shanxi Medical University, Ministry of Education, Shanxi Province, China.

**Keywords:** arrhythmia, cardiomyopathy, heart failure, hypertension, mechanism, myocardial infarction, sodium-glucose cotransporter 2 inhibitor

## Abstract

Sodium-glucose cotransporter 2 inhibitor (SGLT2I) is a new type of hypoglycemic drug that targets the kidney. As research continues to advance on this topic, it has been found that SGLT2I has multiple protective effects, such as hypoglycemic, cardio-renal protective, antihypertensive, and lipid-lowering effects. This review discusses the current concepts and possible mechanisms of SGLT2I in the treatment of heart failure, myocardial infarction, hypertension, cardiomyopathy and arrhythmia to provide a reference for clinicians to use drugs more reasonably and scientifically.

## 1. Introduction

According to the 9th edition of the Global Diabetes Atlas (International Diabetes Federation Diabetes Atlas) published by the International Diabetes Federation in 2019,^[1]^ approximately 463 million adults worldwide have been found to be associated with a risk of diabetes as of 2019. Sixty-eight percent of people with type 2 diabetes (T2DM) die from cardiovascular and cerebrovascular accidents.^[2]^ Therefore, it is important to actively prevent and treat cardiovascular complications throughout the duration of therapy. Despite their glucose-lowering efficacy, traditional hypoglycemic drugs rarely offer cardiovascular protective benefits. In 2008, 1 type of insulin sensitizer (known as rosiglitazone) was delisted due to an increased risk of heart failure or cardiovascular mortality risk.^[3]^ Since then, the US Food and Drug Administration has made it compulsory to conduct cardiovascular outcome studies for all new glucose-lowering drugs. Currently, only a few preclinical trials have been reported to confirm the acute cardio-protective potential of metformin in acute myocardial infarction.^[4,5]^

Sodium-glucose cotransporter 2 inhibitor (SGLT2I) is a new class of glucose-lowering drug and includes drugs such as dapagliflozin, empagliflozin, canagliflozin, ertugliflozin and sotagliflozin, which have already been listed in some countries. This class of drugs improves glycemic control by lowering the renal threshold for glucose, selectively blocking reabsorption of glucose by the kidney proximal convoluted tubule and increasing urinary glucose.^[6]^ Studies have shown that SGLT2I has significantly greater effects in reducing glycated hemoglobin (glycated hemoglobin), body weight and systolic blood pressure (SBP) without adversely affecting cardiovascular safety, whether it is used alone or in combination with other drugs.^[7]^

As more in-depth studies have been conducted, its cardiovascular protection effects have been confirmed. In this context, SGLT2I, which has both hypoglycemic and cardiovascular benefits, has been gradually considered for multiple disease management. In this review, we summarize the latest research on SGLT2I and describe its effectiveness in the treatment of cardiovascular diseases.

## 2. SGLT2I in the treatment of heart failure

### 2.1. Chronic heart failure

#### 2.1.1. Heart failure with reduced ejection fraction (HFrEF).

The dapagliflozin-heart failure study^[8]^ was initiated in February 2017 and showed that for patients with overall HFrEF, cardiovascular mortality or readmission rate for heart failure was reduced by 26% in the dapagliflozin group and by up to 27% in the nondiabetic heart failure population, thus indicating that dapagliflozin can reduce the incidence of major adverse cardiac events (MACEs) in patients, whether they had combined diabetes or not. The EMPEROR-Reduced study^[9]^ was initiated in March of the same year and enrolled patients with worse levels of cardiac function markers than those with dapagliflozin-heart failure, and the results confirmed a 30% reduction in cardiovascular death or readmission rate for worsening heart failure, a 8% reduction in the risk of cardiovascular death and a 31% reduction in first admissions for heart failure in the empagliflozin group compared with the control cohort. The results of these 2 trials certainly provide new options for the treatment of patients with HFrEF.

#### 2.1.2. Heart failure with preserved ejection fraction (HFpEF).

DECLARE-TIMI58 was a multicenter, randomized, double-blind, placebo-controlled trial^[10]^ that enrolled 17,160 patients with T2DM. A total of 1316 of these patients had HFpEF and had a history of or risk factors for cardiovascular disease. The median follow-up time was 4.2 years. A subgroup analysis^[11]^ of this study showed that dapagliflozin demonstrated a significant benefit in the subgroup of HFpEF patients >45%. The EMPEROR-Preserved study^[12]^ included a total of 5988 adult patients with diastolic heart failure (left ventricular ejection fraction [LVEF] >40%), and the median follow-up time was set at 26.2 months. The risk of the composite endpoint event of cardiovascular death or first HF hospitalization was significantly lower in experimental subjects with empagliflozin than in the placebo group, with a reduction of 21%. The EMPEROR-Preserved trial was the first trial to meet its primary end point in patients with symptomatic HFpEF, and it offers hope for patients with this type of heart failure. Moreover, the DELIVER study^[13]^ was performed to verify whether the success of empagliflozin could be reproduced by dapagliflozin in patients with HFpEF. It enrolled NYHA class II to IV in 6263 patients with HFpEF (LVEF > 40% and with structural heart disease). This study is still ongoing, and the results are expected to be available in 2022, which is highly anticipated in the medical community.

### 2.2. Acute decompensation of chronic heart failure

The SOLOIST-WHF trial^[14]^ was a large, randomized, controlled, double-blind trial to investigate the efficacy of SGLT2I for patients with acute decompensation of chronic HF. A total of 1222 adults with T2DM who were admitted for HF and who were treated with intravenous diuretics were enrolled, and 79.1% of the patients had LVEF < 50%. The median follow-up time was 9.2 months. In addition, the primary endpoint was the total number of hospitalizations for cardiovascular reasons and deaths due to emergency visits for heart failure (first and subsequent events). The results showed that sotagliflozin reduced the risk of major events by 33% compared with placebo, with a cardiovascular mortality of 10.6% in the sotagliflozin group and 12.5% in the placebo group. Additionally, there was an all-cause mortality of 13.5% in the sotagliflozin group and 16.3% in the placebo group, as well as an all-cause mortality of 13.5% in the sotagliflozin group and 16.3% in the placebo group (HR = 0.82, 95% CI: 0.59–1.14). This study further expands the indications for SGLT2I for the treatment of heart failure.

A Japanese retrospective study^[15]^ included 31 patients who were admitted to the hospital on an emergency basis with obvious clinical signs or symptoms of heart failure; 12 patients were treated with SGLT2I (9 patients with empagliflozin and 3 patients with canagliflozin), and the remaining 19 patients were treated for conventional acute heart failure. The results demonstrated that both groups recovered to the same extent within the same time frame, but both the use rate of diuretics and aldosterone receptor antagonists were lower in the SGLT2I group during hospitalization, and the dose of the administered diuretic after discharge was also less than in the conventional group. In addition, the SGLT2I group exhibited a significantly higher survival of no AKI (acute kidney injury) than the standard care group, which indicates that treatment with SGLT2I in the early stage of heart failure may reduce the risk of AKI, as AKI is a prognostic marker in cardiovascular disease.^[16]^ Additionally, several other trials on the combinational application of SGLT2I and loop diuretics to heart failure provide a basis for further research in the clinic. Moreover, Christopher S. Wilcox et al selected 42 healthy adults, and the subjects were divided into a bumetanide group (1 mg qd), an empagliflozin group (10 mg qd) and a group with both drugs under the premise of controlled daily sodium intake. After 7 days of observation, the combination group demonstrated a synergistic effect in induced diuresis and natriuresis of mutual adaptation and reversed bumetanide-induced hyperuricemia.^[17]^ Furthermore, Matthew Griffin et al randomized 10 mg qd empagliflozin and placebo in 20 subjects who had heart failure with T2DM and found that empagliflozin caused significant sodium excretion, thus resulting in improved blood volume; additionally, no worsening of potassium loss, renal insufficiency or neurohormonal activation was observed in the 14-day study.^[18]^ Loop diuretics have been considered to potentially increase the incidence of AKI, and the combination with SGLT2I can decrease the dosage of loop diuretics under the premise of decreasing cardiac preload, which helps to reduce the incidence of AKI. In clinical practice, loop diuretics are mostly used in combination with thiazide diuretics to combat diuretic resistance, which results in fatal electrolyte disorders and a deterioration of renal function, and the addition of SGLT2I may prevent this outcome, thus reducing heart failure readmission rates. In conclusion, the efficacy and mechanism of SGLT2I in combination with diuretics still needs to be confirmed by a longer observation period and a wider experimental population.

### 2.3. Heart failure after acute myocardial infarction

The EMMY trial investigated the effect of empagliflozin on cardiac function and markers of heart failure after AMI.^[19]^ In a previous study, an approximate 50% reduction in NT-proBNP levels within 6 months after AMI was confirmed^[20]^; therefore, the primary endpoint of this study was set to reduce NT-proBNP levels. A total of 476 patients with AMI were enrolled. The underlying conditions were creatine kinase >800 U/L and cTnT/cTnI above 10 times the upper limit of normal levels for any of the following factors: significant symptoms of myocardial ischemia, new electrocardiographic myocardial ischemic changes or imaging showing new segmental ventricular wall motion abnormalities. The follow-up period is planned to be 26 weeks, and the study is still ongoing. To date, the use of SGLT2I after cardiovascular events has still been cautiously implemented, although some countries have written SGLT2I into their guidelines. However, uncertainty remains as to whether the drug can be safely used in this setting of heart failure after AMI due to a lack of data, and the completion of the EMMY trial will address this knowledge gap. An experiment in nondiabetic rats with left ventricular dysfunction after myocardial infarction^[21]^ demonstrated a higher ejection fraction in both the early and late treatment groups of empagliflozin than in the control group, and significant improvements were observed in increases in cardiometabolic and ATP content. Thus, SGLT2I may become an effective treatment strategy for heart failure after AMI.

There are several possible mechanisms regarding the treatment of heart failure with SGLT2I. For example, mechanisms can include reductions in cardiac anteroposterior load,^[22]^ increases in endogenous ketone body utilization and the modulation of myocardial energy metabolism,^[23]^ the inhibition of sodium-hydrogen exchange (thereby increasing mitochondrial Ca^2+^ concentrations),^[24]^ the attenuation of epicardial fat deposition,^[25]^ the inhibition of myocardial fibrosis and oxidative stress^[26]^ and the modulation of uric acid^[26]^ and lipid levels,^[27]^ among other mechanisms. In addition, in a 12-month-long clinical trial,^[28]^ left ventricular mass was reduced after the use of dapagliflozin (−3.95 ± 4.85 g in the *experimental* group and −1.13 ± 4.55 g in the placebo group; *P* = .018). Elevated left ventricular mass is closely associated with serious adverse cardiovascular events, heart failure and cardiovascular death^[29]^ and can be an important index for assessing left ventricular remodeling. This study suggests that SGLT2I can reverse left ventricular remodeling, and the reasons may be related to its effect of lowering of body weight and SBP, as well as the improvement of insulin resistance.

SGLT2I breaks the traditional golden triangle treatment of heart failure and adds new clinical evidence. The 2021 American Diabetes Association guidelines recommended SGLT2I as the standard of care for patients with T2DM with atherosclerotic cardiovascular disease (ASCVD) or patients who are considered to be high-risk, as well as those with heart failure and chronic kidney disease, regardless of glycated hemoglobin compliance.^[30]^ The 2021 European Society of Cardiology Heart Failure Congress updated the drug treatment section of the 2021 ESC Guidelines for the diagnosis and treatment of acute and chronic heart failure, which recommends a “new quadruple therapies” of ACEI/ARNI, BB, MRA, and SGLT2I for the treatment of HFpEF.^[31]^

## 3. SGLT2I in the treatment of myocardial infarction

### 3.1. Old myocardial infarction

A subgroup analysis of the DECLARE TIMI-58 trial^[32]^ included 3584 patients with a history of old myocardial infarction and 13,576 patients without a history of myocardial infarction (of whom 3390 patients had a comorbid ASCVD, and 10,186 patients had a comorbid multiple risk factor). Among patients with a history of old myocardial infarction, the cluster of patients who took dapagliflozin had a 2.6% lower chance of MACE events than nonusers, a 16% reduction in relative risk and a 2.6% reduction in absolute risk. There was no significant reduction in the incidence of MACEs in patients with a history of ASCVD but no history of myocardial infarction, and there was no effect in patients without a history of myocardial infarction. The incidence of recurrent myocardial infarction and type 2 myocardial infarction was also found to be significantly reduced in this study. The treatment of the etiology for type 2 infarction is now particularly important; conventional thrombolytic anticoagulation commonly used in type 1 infarctions has limited utility in type 2 infarctions, and an important role for SGLT2I is highly anticipated.

### 3.2. Acute myocardial infarction

Ven G. Lim et al selected diabetic and nondiabetic rats after induction of myocardial infarction caused by the ligation of the coronary artery and randomly divided them into a canagliflozin group and a placebo group for 4 weeks. It was found that canagliflozin resulted in a significant reduction in the area of myocardial infarction compared with placebo, possibly via the activation of the JAK/STAT3 pathway or the AMPK pathway, and the effect was independent of blood glucose levels.^[33]^ Moreover, Masashi Mizuno et al investigated the effect of empagliflozin on cardiomyocyte ultrastructure in the noninfarcted region of the heart after myocardial infarction in diabetic rats. The results showed that empagliflozin normalized the size and number of diabetic cardiac mitochondria and prevented excessive reduction in mitochondrial volume after diabetic myocardial infarction by inhibiting reactive oxygen species clusters and restoring autophagy.^[34]^ The Embody experiment explored the effects of empagliflozin on cardiac sympathetic nerve activity in patients with AMI combined with T2DM and found that both sympathetic and parasympathetic activities were greatly improved following treatment with empagliflozin.^[35]^ In addition, SGLT2I was found to activate the AMPK pathway,^[36–38]^ and AMPK pathway activation attenuates oxidative stress, induces autophagy and promotes mitochondrial biogenesis, thereby antagonizing myocardial injury^[39]^ and exerting a protective effect against myocardial infarction.

These studies demonstrate the protective function and possible mechanisms of SGLT2I for myocardial infarction, thus suggesting that the early initiation of SGLT2I during hospitalization in patients with AMI is expected to be an important target for therapy.

## 4. SGLT2I in the treatment of hypertension

Hypertension is associated with the risk of cardiovascular disease and all-cause mortality, and age at onset is negatively associated with this relationship.^[40]^ A Japanese clinical trial^[41]^ explored the antihypertensive effect of empagliflozin in patients with T2DM combined with nocturnal uncontrolled hypertension on the basis of ARB and showed that empagliflozin was effective in reducing blood pressure during daytime and during nocturnal and 24-hour ambulatory periods. A clinical trial by Eirini Papadopoulou et al demonstrated greater reductions in 24 h brachial SBP (−5.8 ± 9.5 vs −0.1 ± 8.7, *P* = .005) and central SBP (−4.1 ± 8.0 vs −0.7 ± 7.8, *P* = .046) in the dapagliflozin group than in the placebo group in patients with T2DM.^[42]^ Furthermore, Alexander J.M. Brown et al found that dapagliflozin significantly reduced 24-hour ambulatory and nocturnal SBP.^[28]^ The disappearance of the nocturnal drop in blood pressure has been identified as an important marker of cardiovascular risk (independent of overall blood pressure over 24 hours), which further demonstrates the cardiovascular protective effects of SGLT2I.

Possible antihypertensive mechanisms include SGLT2I reducing blood volume in patients via metabolic and hemodynamic pathways and having a significant antihypertensive effect in patients with volume-dependent hypertension. In addition, SGLT2I suppresses the renin-angiotensin system and reduces angiotensin II.^[43]^ In previous clinical trials, SGLT2I was found to lower blood pressure without compensatory heart rate acceleration, and subsequent studies in rats with metabolic syndrome demonstrated its ability to ameliorate the effects of circadian rhythms of the sympathetic nervous system^[44]^ and a significant sympathetic depressive effect (mainly during sleep).^[45]^ Furthermore, Hotimah et al found that ipragliflozin attenuated vascular endothelial function impairment in mice,^[46]^ thus confirming the benefit of SGLT2I in another hypertensive manner.

## 5. SGLT2I in the treatment of cardiomyopathy

Diabetic cardiomyopathy is often secondary to metabolic injury, the core factors of which include insulin resistance and abnormalities in the internal environment due to disturbances in body glucolipid metabolism. Myocardial fibrosis is an important pathological change regarding its progression, for which there is a lack of specific therapeutic approaches. In an animal experiment conducted by M. Arow et al ^[47]^ using mice with diabetic cardiomyopathy induced by angiotensin II, it was found that dapagliflozin could apparently improve myocardial fibrosis, reduce the inflammatory response and decrease reactive oxygen species production. Second, dapagliflozin caused a reduction in the magnitude of calcium transients and area under the curve in cardiomyocytes, thereby preventing intracellular calcium overload, increasing the mitochondrial uptake of calcium and enhancing myocardial contraction. Isolated experiments revealed that this alteration did not correlate with blood glucose values. This study provides direction for SGLT2I in the treatment of cardiomyopathy and further reveals the mechanism of its cardiovascular protection. Several other experiments have also demonstrated the inhibition of myocardial fibrosis via SGLT2I.^[26,48]^ For example, Tsung-Ming Lee et al suggested that SGLT2I enhanced M2 macrophage activation, reduced myofibroblast infiltration and attenuated myocardial fibrosis in postinfarction rats by regulating the macrophage phenotype through a (RONS)/STAT3-dependent pathway.^[49]^

## 6. SGLT2I in the treatment of arrhythmias

A meta-analysis demonstrated a 18% reduction in the risk of atrial fibrillation (AF) and a 27% reduction in the risk of ventricular tachycardia after using SGLT2I.^[50]^ Another meta-analysis also demonstrated a 19.33% reduction in the incidence of serious adverse events in AF/atrial flutter. The lowest incidence of serious adverse events in AF/atrial flutter was treated with dapagliflozin compared with other drugs in the same family.^[51]^ However, a meta-analysis^[52]^ involving ventricular arrhythmias (VAs, including ventricular tachycardia and ventricular fibrillation) showed no association between SGLT2I treatment and the risk of VAs (risk ratio = 0.84, 95% CI: 0.66–1.06; *P* = .14), yet a subgroup analysis showed that low-dose SGLT2I treatment resulted in a reduction in VAs compared with control or placebo groups. In response to this result, the authors found that left ventricular volume can indicate whether adverse ventricular remodeling has occurred in patients with heart failure,^[53]^ whereas a meta-analysis showed that SGLT2I treatment had little effect on left ventricular end-diastolic volume and LVEF.^[53]^ Its effect on adverse ventricular remodeling was mild; therefore, its anti-VAS potential was small. Second, the small sample size included in this study resulted in a wide confidence interval, which indicated an inaccurate result. In conclusion, the benefit of SGLT2I in the treatment of AF is positive,^[54]^ whereas its protective effect on VAs and the long-term effects of arrhythmias remain inconclusive and need to be confirmed by further clinical trials.

The mechanisms of SGLT2I for AF are still unclear. In addition to the cardioprotective mechanisms mentioned above, 1 of the other possible reasons is that diabetes is known to exacerbate atrial remodeling, thereby promoting the onset and progression of AF.^[55]^ Abnormalities in mitochondrial structure and function also contribute to atrial structural and electrical remodeling.^[56]^ The diabetic rat experiment by Qingmiao Shao et al^[57]^ found that empagliflozin significantly improved the respiratory function and membrane potential of atrial mitochondria, impaired mitochondrial biogenesis and inhibited atrial remodeling and myocardial interstitial fibrosis, thereby reducing the incidence of AF. Moreover, inflammatory responses are thought to be associated with the development of AF, and AF also promotes increased levels of inflammatory markers,^[58]^ thus resulting in a vicious cycle, whereas several experiments have demonstrated the inhibition of microvascular inflammation and the reduction of oxidative stress by SGLT2I.^[47,59]^ In addition, SGLT2I activates the AMPK pathway (as mentioned above), and there are several experiments that have confirmed the benefit of this pathway activation for AF treatment.^[60,61]^ The suppression of AF by SGLT2I will further expand its indications.

## 7. Conclusion

SGLT2I is currently occupying an increasingly important position due to its hypoglycemic, renal and cardiovascular protective functions. Its efficacy and safety still need to be further explored. It is important to consider SGLT2I for the integrated management of multiple risk factors and protection of patients’ integrity, and this drug has a promising future.

## Author contributions

**Project administration:** Qinghua Han.

Writing – original draft: Yingxia Zhang.

Writing – review & editing: Yingxia Zhang.
